# RNA sequencing reveals a complete but an unconventional type of dosage compensation in the domestic silkworm *Bombyx mori*

**DOI:** 10.1098/rsos.170261

**Published:** 2017-07-12

**Authors:** Gajula Gopinath, Kuchi Srikeerthana, Archana Tomar, Srikakolapu M. Ch. Sekhar, Kallare P. Arunkumar

**Affiliations:** Centre of Excellence for Genetics and Genomics of Silkmoths, Laboratory of Molecular Genetics, Centre for DNA Fingerprinting and Diagnostics, Hyderabad 500001, India

**Keywords:** female heterogamety, lepidopterans, Z-linked expression, dosage compensation, *Bombyx mori*

## Abstract

Sex chromosomal dose difference between sexes is often normalized by a gene regulatory mechanism called dosage compensation (DC). Studies indicate that DC mechanisms are generally effective in XY rather than ZW systems. However, DC studies in lepidopterans (ZW system) gave bewildering results. In *Manduca sexta*, DC was complete and in *Plodia interpunctella*, it was incomplete. In *Heliconius* species, dosage was found to be partly incomplete. In domesticated silkmoth *Bombyx mori*, DC studies have yielded contradictory results thus far, showing incomplete DC based on microarray data and a possible existence of DC based on recent reanalysis of same data. In this study, analysis of *B. mori* sexed embryos (78, 96 and 120 h) and larval heads using RNA sequencing suggest an onset of DC at 120 h. The average Z-linked expression is substantially less than autosomes, and the male-biased Z-linked expression observed at initial stages (78 and 96 h) gets almost compensated at 120 h embryonic stage and perfectly compensated in heads. Based on these findings, we suggest a complete but an unconventional type of DC, which may be achieved by reduced Z-linked expression in males (ZZ). To our knowledge, this is the first next-generation sequencing report showing DC in *B. mori*, clarifying the previous contradictions.

## 1. Introduction

Sex chromosomes are believed to have evolved from autosomes through intermediate proto-sex chromosomes. The evolution of sex chromosome is thought to be initiated, once a chromosome from a pair acquires the sex-determination gene [1]. In the process of acquiring sex-determination function [2], the accumulation of sexually antagonistic mutations and repeat elements by Y (W) would have mostly contributed for the loss of homology with X/Z. Such loss of homology is believed to be the driving force for Y (W) recombination isolation [3] and its degeneration via gene loss [4], thus leaving exclusive sex-determination function [5]. Thus, the evolution of Y for establishing two distinct sexes, males (XY) and females (XX), had resulted in ‘X chromosome aneuploidy’ [6]. This aneuploidy creates the dose difference in X-linked genes, which could be deleterious if not compensated at the level of expression. Hence, organisms have adopted a versatile gene regulatory mechanism called dosage compensation (DC), which is limited mostly to homogametic sex (X/Z) chromosomes in most species [7].

Reports from flies to mammals suggest diverse epigenetic mechanisms for the phenomenon of DC in several male heterogametic species (XX/XY). In *Drosophila melanogaster*, the DC is achieved by direct upregulation of entire X-linked genes, selectively in males (XY) [8,9]. In *Caenorhabditis elegans* and mammals, DC has been proposed to have evolved in a two-step process (Ohno's hypothesis)[10–14]. First, there is a transcriptional upregulation of the X-linked genes in both sexes. Second, there is a downregulation of all the X-linked genes in female sex (XX) to rescue from the detrimental effects of hypertranscription [10–14]. In *C. elegans*, there is a transcriptional upregulation in males (XO) and hermaphrodites (XX) [15], whereas in hermaphrodites an additional mechanism of transcriptional repression operates to rescue them from the deleterious effects of hyperexpression of XX [16–18]. In the case of mammals, there is a phenomenon of X inactivation in females; thus, the single X in both sexes are upregulated to match the level of autosomes [15,19,20]. In beetles, there is an upregulation of X-linked genes in both males (XY) and females (XX) with no downregulation of XX in female sex [21]. This leads to hyperexpression of XX in females [21]. But this hyperexpression in females has been challenged recently [22], suggesting the existence of a proper DC in beetles. In *Anopheles stephensi*, DC was found to be complete [23] with equally expressing X-linked loci between sexes, whereas in *A. gambiae* it is incomplete [24].

The patterns of DC in XX/XY species suggest that the mechanism of DC operates in a chromosome-wide manner and has evolved not only to (i) equalize the expression of sex chromosomes between sexes (first condition, X_male_ = X_female_) but also (ii) to abolish the expression disparity between autosomes and sex chromosomes in both sexes (second condition, X = A) [20]. As these two conditions were consistently observed in XX/XY species with DC, they have become the evolutionary precedent. Hence, DC is viewed as complete and conventional, only when both these conditions are fulfilled in an organism. The first condition of DC is believed to surpass the deleterious effects of sex chromosome aneuploidy [25], and it appears to be fundamentally right as it implies the necessity of DC in a species. By contrast, the second condition of complete DC must have been set based on two things: (i) insights from sex chromosome evolution [11,26] and (ii) the general pattern of equally expressing autosomal and X-linked genes, in most of the male heterogametic species (XX/XY) studied for DC. Thus, the second condition of complete DC appears to be more instinctively assigned. Following this assumption, the ancestral average expression of proto-X or proto-Z need not be necessarily equal to that of autosomes, as even the individual autosomes differ at their average expression levels. Indeed, the ancestral expression of proto-X or proto-Z will be determined by the gene constitution and their expression profile. Hence, this ancestral expression of proto-X or proto-Z is a crucial factor in determining the path of DC evolution in a species.

Furthermore, as the patterns of DC are highly variable, evolved independently and dynamic across sex-determination systems and species [7,13,14], a variety of approaches with a high degree of flexibility are anticipated for attaining ‘DC’ in a species. These approaches may not be following the evolutionary precedent of XX/XY system, for the approval of the existence of DC in a species. Based on these arguments, a species can be considered to have DC and fulfil the primary objective of DC if it satisfies the ‘first condition’. As the ‘second condition’ of complete DC is set by the evolutionary precedent, it may be limited to XX/XY systems and may not be universally applicable and readily extrapolated to female heterogametic systems like ZZ/ZW or ZZ/ZO.

Among female heterogametic species (ZZ/ZW), DC was first assessed in chicken [27,28] and is found to be ineffective as the Z-linked genes showed approximately 1.4-fold higher expression in males. This was accompanied by similar results from *Bombyx mori*, a lepidopteran [29] and a trematode parasite, *Schistosoma mansoi* [30], giving an impression that DC is generally absent or incomplete in a female heterogametic (ZZ/ZW) system. However, recent genome-wide studies based on RNA sequencing (RNA-seq) in ZZ/ZW systems have yielded an unexpected result of complete DC in a lepidopteran, *Manduca sexta*, with equally expressing Z-linked genes between sexes and with an expression parity between autosomal and Z-linked genes [31]. This has renewed much interest in the phenomenon of DC in ZZ/ZW species. However, in another lepidopteran species *Plodia interpunctella* [32], DC was demonstrated to be incomplete as it showed just over half of average expression of female Z-linked genes to that of males. Recently, in *Heliconius* (butterflies), the DC was reported to be imperfect or not complete, due to a consistent male-biased expression of Z-linked genes in various tissues tested [33]. Thus, a mixed pattern of DC mechanisms can be observed in female heterogametic species.

DC in female heterogametic species is proposed to be specified to a subset of dose-sensitive Z-linked genes (incomplete DC; [13]) and in a few others, it may be operating in a chromosome-wide manner [31]. In addition, the ZZ/ZW systems would have adapted many auxiliary phenomena for assisting the primary objective of DC, for example, the enrichment of the male-biased genes on the Z chromosome in a few female heterogametic species [34–36], which lead to sex chromosome-biased expression (SCBE) of a set of Z-linked genes. The female heterogametic species may be taking an advantage of SCBE. One striking example would be the sex determination in ZZ/ZO system (in wild silkmoths, *Antheraea assama* and *Ant. mylita*), where ZZ are males and ZO are females. Here, the double dose of Z chromosome stands as an essential criterion for male determination, presumably due to the male-biased expression of at least a subset of Z-linked genes. Thus, in this aspect, the sex-biased expression observed in most of the ZW species may be a general norm and not a disability; moreover, their successful survival and proliferation supports this assumption. Thus, the homogametic sex chromosomes (X/Z) were considered to be highly influenced by two phenomena: (i) the SCBE and (ii) the DC mechanism. It is not very clear whether SCBE and DC have coevolved or DC is followed by SCBE or vice versa [37]. The SCBE of X (Z) chromosome, achieved by an ‘in and out’ gene trafficking [38,39], may strengthen the sexually dimorphic traits; besides, this can be perceived as a counter-attacking force of DC.

The initial reports for DC in *B. mori* based on the expression profile of a set of a few Z-linked genes have suggested an incomplete DC, where males showed higher expression of Z-linked genes [40,41]. This was further confirmed by global, microarray analysis [29]. However, another study using reanalysis of the same microarray data draws a clue for the possibility of a globally operating DC mechanism in *B. mori* [42]. RNA-seq was proved to be an efficient tool in addressing DC on a genome-wide scale [23,24,31–33]. There are two recent reports (based on RNA-seq), one for the involvement of a Z-linked gene called *masc* in *B. mori* DC [43] and another for the existence of an incomplete dosage in very early stage, and most of the Z-linked genes are dosage-compensated by 78 h of development [44]. Furthermore, in *Bombyx*, the process of sex determination is governed by the differential splicing of *doublesex* gene, *Bmdsx.* It has two splicing isoforms, *Bmdsxf* in females and *Bmdsxm* in males. These isoforms produce differential proteins, having antagonistic functions in sexes thus inducing sexual differentiation. Based on the *Bmdsx* splicing in eggs at various developmental stages, it is found that *Bmdsxf* splicing isoform is predominant at 12 h of development. This could be due to the maternal deposition of *Bmdsxf* pre-mRNA [45]. In the same study, at 24 h of development, there is a shift of splicing from female to male form, indicating the endogenous expression of *Bmdsx* mRNA [45]. However, in this study, few eggs showed an equal expression of *Bmdsxf* and *Bmdsxm* isoforms; probably these are female eggs. As the development progresses, there is a shift in splicing from the predominant or equally expressing *Bmdsxm* isoform to *Bmdsxf* isoform significantly in female eggs between 29 and 32 h. Based on these results, it was determined that the sex of the embryo gets fixed in between these developmental time points (29–32 h) [45]. However, this analysis was done in non-diapause strains [45].

**Figure 1.**
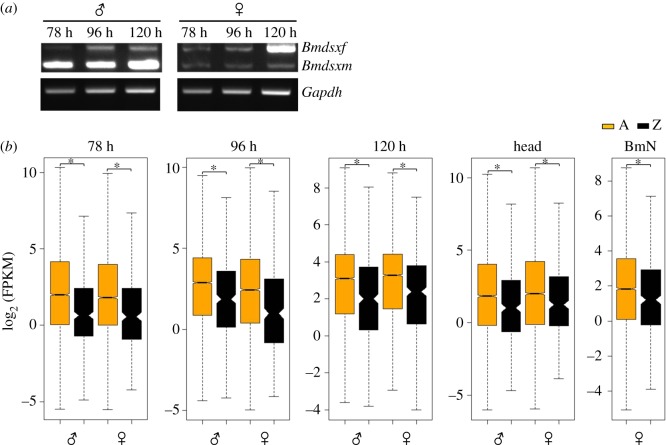
(*a*) The sex-specific differential splicing pattern of *Bmdsx* in the embryonic stages of 78, 96 and 120 h. (*b*) Box plot of the log_2_-transformed FPKM distribution of autosomal (A, yellow) and Z-linked (Z, black) genes that showed expression (FPKM ≠ 0) in male and female samples. The boxes represent the interquartile range (IQR), the notch of box plots represents median (horizontal line) expression with a 95% confidence interval and the outliers were not plotted. The asterisk represents a significantly (MWU, *p* < 0.0001) reduced expression of Z-linked genes when compared with that of the autosomes.

In the current study, we did RNA-seq for three different embryonic stages of a diapausing bivoltine strain (78, 96 and 120 h) and fifth-instar larval heads. The relative expression of Z-linked genes to that of autosomal (Z : A) and the Z-linked genes between sexes (male : female, M : F) were analysed between male and female samples. In bivoltine strain, the eggs undergo diapause and are not hatched in 10 days. Hence, the eggs have to be acid-treated to break the diapause. It is also known that the development of these eggs is comparatively slower than the non-diapause eggs; hence, there is a possibility of delayed development in the eggs. In diapause eggs, we have found a similar pattern of *Bmdsx* splicing shift in male and female embryos, however, at later stages of development, i.e. at 78, 96 and 120 h. We have used fifth-instar larval heads as a reference sample (DC is expected to be established) for the egg samples. Based on the differential splicing of *Bmdsx*, we consider the 78 and 96 h stages to be before sex-determination stages and 120 h to be after sex-determining stage. Hence, the sex is determined in between the 96 and 120 h stage of development in diapause eggs of *B. mori* (figure 1*a*). However, in both the studies (diapause and non-diapause strains), it was found that it is the differential splicing of *Bmdsx* that occurs first followed by the advent of DC in *B. mori*. From these observations, we infer that though the time points for *Bmdsx* differential splicing and DC vary between non-diapause and diapause strains, the patterns of the occurrence of these processes are sequential and are comparably similar.

## 2. Results

### 2.1. Read mapping and sequencing output

RNA-seq of three sexed embryonic stages (78, 96 and 120 h), fifth-instar larval heads and BmN cells of *B. mori* resulted in 718 M reads (359 M paired) of 100 bp length from nine samples ranging from 66 to 126 M paired-end reads (electronic supplementary material, table S1). The use of the Trimmomatic software for the trimming of the adapter sequences resulted in 666 M reads (333 M paired). Except for the head samples, the technical replicates (duplicates) were included for all the samples. The Fragments Per Kilobase of transcript per Million mapped reads (FPKM) values for each gene were averaged across the technical replicates and used for analysis. Genome-guided mapping using Bowtie (v. 2.1.0) resulted in an average of 78.9% read alignment per sample (electronic supplementary material, table S1) of the quality filtered and adapter trimmed reads. Based on the scaffold identity, the genes were grouped into autosomal (A)- and Z-linked (Z) genes. The unmapped genes were excluded from further analysis. Similar to a conventional dosage analysis, we have tested dosage in *B. mori* by two estimates: (i) Z : A ratios (autosomal relative expression of Z-linked genes) and (ii) M : F ratios (sex-biased expression of A- and Z-linked gene expressions).

### 2.2. Z : A ratios: relative expression of Z chromosome

**Figure 2.**
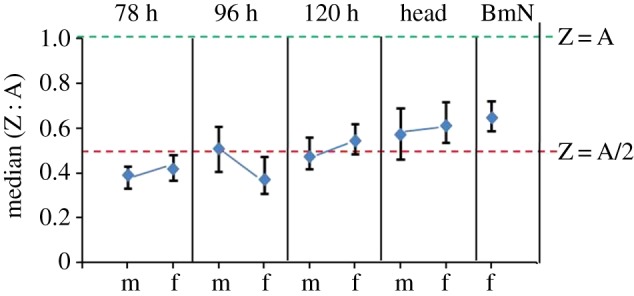
The bootstrapped median of autosomes to Z chromosome (Z : A) gene expression ratios for the samples. The dashed lines (green and red) correspond to the relative expression of Z to that of autosomes. The ratio of Z : A of 1 and 0.5 indicates an equal (Z = A) and half expression (Z = A/2) of Z chromosome to that of autosomes, respectively. m, male; f , female. Error bars represent the 95% confidence intervals for the median estimated from 10 000 bootstrap replicates.

**Figure 3.**
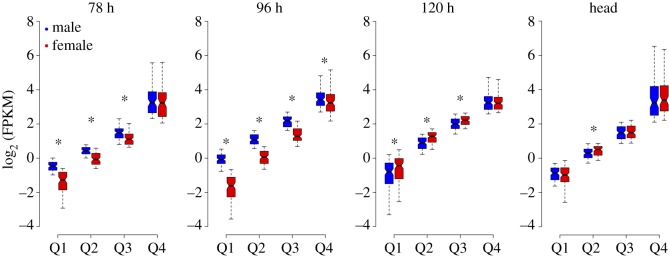
Comparison of quartile expressions for Z-linked genes dataset used for Z : A analysis (figure 1*b*). The log_2_ FPKM expression data for the Z-linked genes were segregated as quartiles based on independent binning. The boxes represent the interquartile range (IQR), the notch of box plots represents median (horizontal line) expression with a 95% confidence interval and the outliers were not plotted. The asterisk indicates a significant difference between quartile expressions (MWU, *p* < 0.05).

The Z : A ratio is informative about how DC is achieved in organisms. It provides an autosomal relative expression of Z-linked genes [13]. The Z : A ratio values of 0.5, 1 and 2 correspond to half, equal and double expression of Z-linked genes, respectively, to that of autosomes. The mean- (using FPKM data) and median-based (using log_2_-transformed FPKM) Z : A ratios were estimated from the ‘true expression dataset’ (FPKM ≠ 0) (figure 1*b* and table 1). Thus, the estimated mean and median Z : A ratios approximately ranged from 0.4 to 0.6 in both sexes, suggesting a significantly low expression of Z-linked genes compared with that of autosomes in all the analysed samples (embryonic stages of 78, 96 and 120 h; larval head and BmN cells). The non-parametric Mann–Whitney *U* tests (MWU; A ≠ Z) statistically support the significant difference observed between A and Z expression levels (figure 1*b* and table 1). At 78 h, the median Z : A ratios for male (0.39) and female (0.42) were substantially lower than 0.5, suggesting an initially less than half expression of Z-linked genes to that of autosomal genes (figure 2 and table 2). At 96 h stage, this ratio was higher in male (0.51) compared with female (0.37) embryos, depicting a relatively (autosomal) increased expression of Z-linked genes in males. This scenario was reversed at 120 h stage where the male (0.47) and female (0.54) ratios indicate a relatively (compared with that of autosomes) increased expression of Z-linked genes in females. These median Z : A ratio profiles of the embryonic samples (78–120 h stages) present a dynamic picture of relative (compared with that of autosomes) expression of Z-linked genes in the process of acquisition of DC (figure 2 and table 2). Head (male = 0.57, female = 0.61) and BmN (0.65) cells showed a median Z : A ratio of approximately 0.6, suggesting a relative just over half expression of Z-linked genes compared with that of autosomes (figure 2 and table 2). We also did a quartile-based analysis for the Z-linked genes (from true expression dataset), and the results were consistent at various magnitudes of expression (figure 3). The point estimate of Z : A ratios and the non-parametric MWU tests together suggest a substantially reduced expression of Z-linked genes to that of autosomal in both sexes.

**Table 1.** The mean and median expression of Z-linked and autosomal loci across various embryonic stages, head and BmN cells of *Bombyx mori*. The mean/median Z : A ratios (italicized) signify the relatively low expressed Z-linked genes to that of autosomal genes. The ratio values of less than 0.5, approximately 0.5 and greater than 0.5 correspond to less than half, half and more than half of average expression of Z-linked genes relative to that of autosomal genes.

	78 h		96 h		120 h		Head		BmN
statistic	male	female	male	female	male	female	male	female	ovary
mean autosomal FPKM	26.34339	32.68268	19.31366	22.9903	18.73881	17.45362	30.64596	30.85957	20.1034
mean Z-linked FPKM	13.55054	16.68408	12.42164	12.30837	11.8242	11.28153	19.1806	19.69835	11.65642
mean Z : A ratio	*0.514381*	*0.510487*	*0.643153*	*0.535372*	*0.631001*	*0.646372*	*0.625877*	*0.638322*	*0.579823*
median autosomal FPKM	3.99718	3.490582	7.354102	5.405826	8.613708	9.700895	3.579668	3.996381	3.569715
median Z-linked FPKM	1.567086	1.480323	3.662872	1.958461	4.0324	5.25235	2.053293	2.389728	2.320983
median Z : A ratio	*0.392048*	*0.424091*	*0.498072*	*0.362287*	*0.468138*	*0.541429*	*0.573599*	*0.597973*	*0.650187*
MWU *p*-value: A ≠ Z-linked	<0.0001	<0.0001	<0.0001	<0.0001	<0.0001	<0.0001	<0.0001	<0.0001	<0.0001
no. of Z-linked loci	399	321	435	379	460	467	382	361	398
no. of A-linked loci	9511	8235	9925	9224	10 125	9990	7825	7841	9248

**Table 2.** The bootstrapped median Z : A ratio values with lower and upper confidence intervals for male and female embryonic, head and BmN cells of *Bombyx mori*.

sample	median	lower—CI	upper—CI
m 78 h	0.389312	0.331252	0.430176
f 78 h	0.418994	0.366021	0.480964
m 96 h	0.50698	0.40669	0.607097
f 96 h	0.370874	0.307573	0.472701
m 120 h	0.474671	0.417544	0.559419
f 120 h	0.542239	0.485654	0.618566
m head	0.570777	0.463294	0.688725
f head	0.609628	0.53812	0.718968
BmN	0.646624	0.587231	0.719966

### 2.3. Male : female ratio distributions—sex-biased expression of Z chromosome

**Figure 4.**
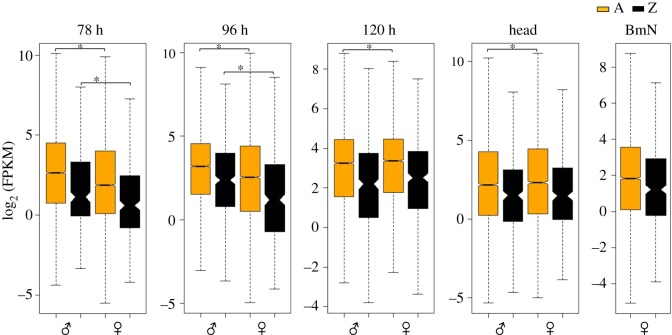
Box plot of the log_2_-transformed FPKM distribution of autosomal (A, yellow) and Z-linked (Z, black) genes that expressed (FPKM ≠ 0) in both male and female samples. The boxes represent the interquartile range (IQR), the notch of box plots represents median (horizontal line) expression with a 95% confidence interval and outliers were not plotted. The data indicate a significant male-biased expression of autosomal and Z-linked genes at the early embryonic stages of 78 and 96 h (MWU, *p* < 0.0001). The asterisk indicates the significant difference observed between male and female autosomal or Z-linked genes. The significant difference observed between the autosomal genes at 120 h embryonic stage (MWU, *p* < 0.05) and head samples (MWU, *p* < 0.01) could be due to the local effects.

**Figure 5.**
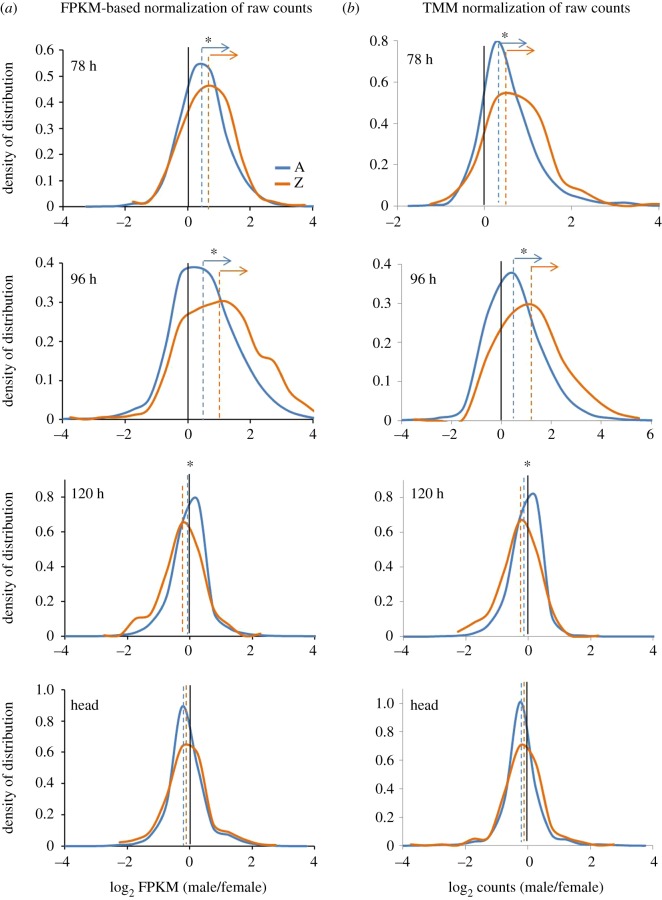
The M : F distributions of autosomal and Z-linked genes in the embryonic (78, 96 and 120 h) and head samples. (*a*) The M : F distributions from FPKM-based normalization of raw counts. (*b*) The M : F distributions from TMM normalization of raw counts. The dashed vertical lines represent the median of the frequency distributions (shown in dashed lines). An asterisk represents the significant difference (MWU, *p* < 0.01) observed between autosomal and Z-linked M : F distributions. At 78 and 96 h stages, the arrows indicate the profound male-biased expression of autosomal (MWU, *p* < 2.2 × 10^−16^) and Z-linked (MWU, 78 h, *p* = 0.0005 and 96 h, *p* = 2.127 × 10^−9^) genes.

The M : F ratio distributions indicate the sex-biased expression of autosomal and Z-linked genes. These distributions are directly compared for testing DC in a species. We performed three kinds of assessments from M : F ratio distributions: (i) sex-biased expression of Z-linked genes, (ii) sex-biased expression of autosomal genes and (iii) the difference in the M : F distributions of A- and Z-linked genes. The Z-linked genes showed a significant male-biased expression at 78 h (MWU, *p* = 0.00056) and 96 h (MWU, *p* = 2.127 × 10^−9^) embryonic stages, whereas such difference was not observed at 120 h (MWU, *p* = 0.08008) embryonic stage and also in head samples (MWU, *p* = 0.5517) (figure 4). Based on these findings, we assume that DC (sex chromosome DC) would have initiated after 96 h and became established at 120 h stage of embryonic development. Interestingly, the male-biased expression of Z-linked genes at 78 h (MWU, *p* = 0.00056) and 96 h (MWU, *p* = 2.127 × 10^−9^) stages is also coupled with a significant male-biased expression of autosomal genes at 78 h (MWU, *p* < 2.2 × 10^−16^) and 96 h (MWU, *p* < 2.2 × 10^−16^) samples. This may be due to the dosage uncompensated effects of Z-linked genes (male biased) at 78 and 96 h stages, which may significantly influence the expression of autosomal genes (figure 5 and table 3). But a significant difference was also observed for the autosomal expressions between sexes at 120 h (MWU, *p* = 0.01172) and head (MWU, *p* = 0.006069) samples where the effect of DC is visible (figure 5 and table 3), and this could be due to the local effects being established by the process of sexual differentiation. For instance, the knockdown of *masc* in female embryos had resulted in upregulation of a few Z-linked genes; this was accompanied by downregulation of a significant number of autosomal genes [43]. This suggests the role of Z-linked genes in influencing the expression of several autosomal genes. Further in *B. mori* Z-linked gene, *masc* controls the differential splicing of *Bmdsx* [43] that regulates sexual dimorphism and differentiation genetic network by influencing the expression of numerous autosomal genes [46]. In a similar way, various Z-linked genes may be globally influencing the expression levels of various autosomal genes.

**Table 3.** The M : F gene expression ratios for Z-linked and autosomal loci in *Bombyx mori* for various embryonic stages and head tissues.

sample	Z-linked loci	autosomal loci	^(a)^ median Z-linked (M : F)	^(b)^ median autosomal (M : F)	^(a÷b)^ Z : A ratio of medians	MWU *p*-values; male Z ≠ female Z	MWU *p*-values; male A ≠ female A	MWU *p*-values; autosomal M : F ≠ Z-linked M : F
78 h	310	8102	1.534561	1.397903	1.097748	0.0005608	<2.2 E-16	0.005881
96 h	365	8983	2.039096	1.359739	1.499623	2.13 E-09	<2.2 E-16	<2.2 E-16
120 h	442	9632	0.884246	0.997452	0.886504	0.08008	0.01172	1.59 E-09
Head	339	7210	0.935571	0.916635	1.020661	0.5517	0.006069	0.4517

**Figure 6.**
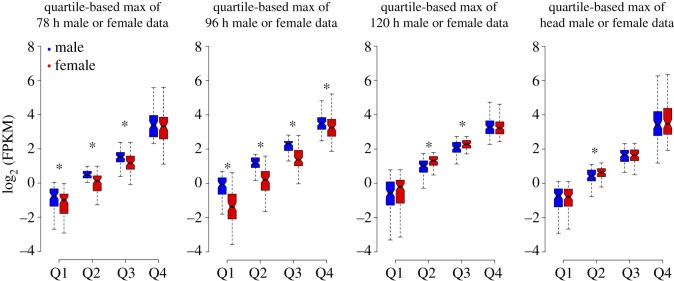
Comparison of quartile expressions for Z-linked genes dataset used for M : F analysis. The log_2_ FPKM expression data for the Z-linked genes were segregated as quartiles based on max of male- or female-paired data. The boxes represent the interquartile range (IQR), the notch of box plots represents median (horizontal line) expression with a 95% confidence interval and the outliers were not plotted. The asterisk indicates a significant difference between quartile expressions (MWU, *p* < 0.05).

Furthermore, we estimated the difference between the M : F distributions for A- and Z-linked genes. We found a significant difference between these distributions at 78 h (MWU, *p* = 0.005881), 96 h (MWU, *p* < 2.2 × 10^−16^) and 120 h (MWU, *p* = 1.588 × 10^−9^) but not for the head samples (MWU, *p* = 0.4517), suggesting that DC is apparent in head sample. The differences in the M : F distribution plots were also reflected in the Z : A ratio of medians (table 3); this ratio at 78 h (1.1) represents a slightly male-biased expression of Z-linked genes and a profoundly male-biased expression at 96 h (1.5); female-biased expression at 120 h (0.89) and an almost unbiased expression in head (1.02) samples (figure 5 and table 3). Additionally, we did quartile-based max of male- or female-paired data analysis [33] for the Z-linked genes from the dataset used for M : F distribution analysis (figure 5) and showed a consistency in the observed biased expressions of Z-linked genes at various magnitudes of gene expression (figure 6).

**Figure 7.**
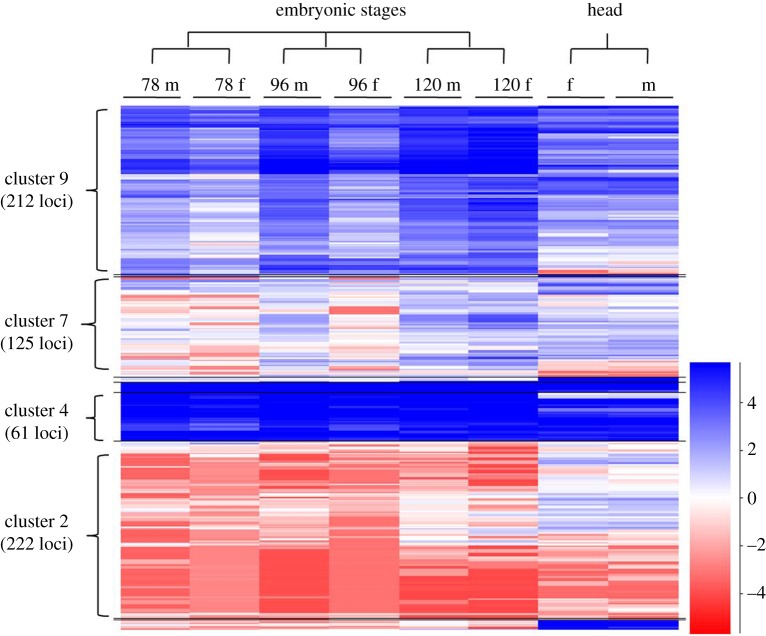
Schematic representation of Z-linked gene expression. The genes mapped to Z-linked scaffolds were segregated and their log_2_ FPKM values were plotted as a heatmap. The heatmap depicts a male-biased cluster 9 (212 loci) with higher expression, a female-biased cluster 2 (222 loci) with lower expression, cluster 7 (125 loci) represents genes with varied expression and an unbiased cluster 4 (61 loci).

The expression of Z-linked genes (log_2_ FPKM) was compared between sexes by using a heatmap (figure 7). The genes with a relatively higher expression level showed a male-biased expression at early developmental stages, 78 and 96 h (cluster 9, 212 genes). The lowly expressing genes showed a female-biased expression (cluster 2, 222 genes), exclusively at 78 and 96 h stages, and also showed an overall male-biased to -unbiased expression at 120 h stage. The cluster of genes having a very high level of expression (cluster 4, 61 genes) showed an almost unbiased expression in all the samples. Almost all the clusters appeared to be compensated by having a similar level of expression between sexes at 120 h stage, except for the mosaic cluster (cluster 7, 125 genes). In the head samples, almost all of the Z-linked genes were found to be dosage-compensated, except for a very few local effects.

**Figure 8.**
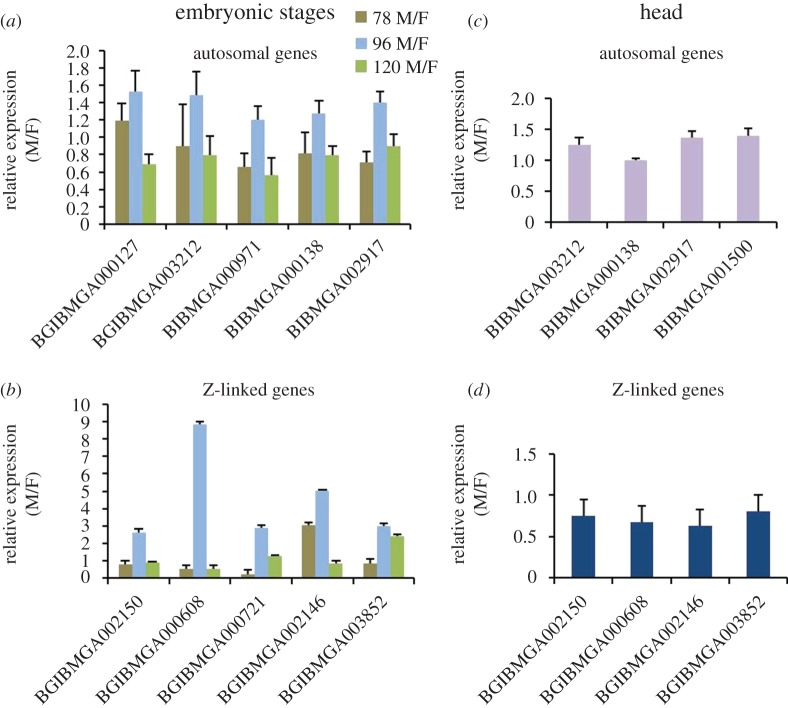
Validation of RNA-seq results of embryonic stages (78, 96 and 120 h) and head samples by qRT-PCR. *rp49* was used as an endogenous reference expression. Female samples were used as calibrators; hence, the relative expression (M/F) of females can be taken as 1 for all the genes. The relative expression of selected five autosomal (*a*) and five Z-linked genes (*b*) for the three embryonic stages and four equally expressing (M/F = approximately 1) autosomal (*c*) and four Z-linked genes (*d*) were shown in the head samples.

Finally, to evaluate the profound male-biased expression at 96 h stage from the embryonic RNA-seq data, we selected and analysed five Z-linked and five autosomal gene expressions from different chromosomal locations through quantitative reverse-transcription PCR (qRT-PCR) using *rp49* as an endogenous control. We chose *rp49*, because it is the best endogenous reference gene, with a least stability index of 0.083 in *B. mori* when compared with other endogenous reference genes like *E2F*, *actinA1*, *actinA3*, *G3PDH* and *GAPDH* [47]. Autosomal genes of embryonic stages showed an overall equal expression in all three stages (figure 8*a*). All the five Z-linked genes showed a relatively higher fold expression at least in 96 h stage (figure 8*b*). For the head samples, the selected four autosomal (figure 8*c*) and four Z-linked genes (figure 8*d*) (based on FPKM values) were shown to be almost equally expressing in both sexes in qRT-PCR. The qRT-PCR data of selected genes support the FPKM-based relative expression from RNA-seq data analysis. Furthermore, we have found that the expression level of *masc* is relatively higher (6.45-fold) in male embryos compared with female embryos (electronic supplementary material, table S2) at 96 h of development, a key gene, having roles in both DC and also in sex determination.

## 3. Discussion

### 3.1. Relatively reduced expression of Z-linked genes compared with that of autosomes

**Figure 9.**
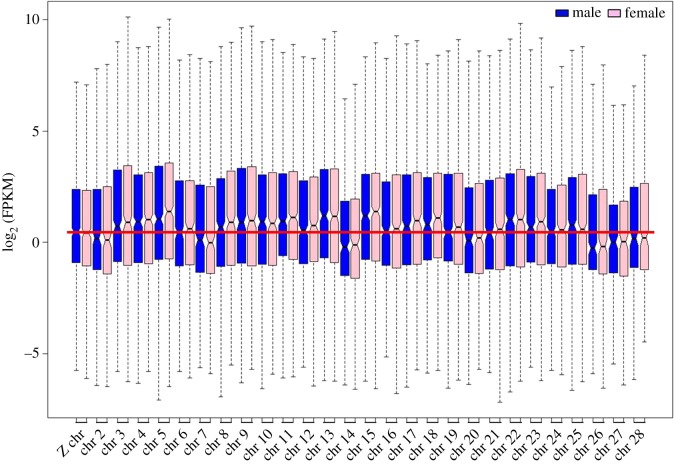
The relative expression of Z chromosome compared with autosomes in the head samples. The solid red line represents the Z chromosome expression level in comparison with autosomes. Z chromosome expression is approximately less than 12, higher than seven and almost equal to nine autosomes. The outliers were not plotted and the notch of the box plots represents the median expression with a 95% confidence interval.

Our study showed that in *B. mori*, Z : A ratio is less than 1 in both the sexes, representing the reduced expression of Z-linked genes to that of autosomes. A plausible explanation for this unusually reduced Z : A ratio is the low expression of Z-linked genes compared with a major proportion of autosomes (12, which is 48.06% of the genome) (figure 9 and electronic supplementary material, table S3). The case of *B. mori* is similar to *Heliconius* species, where the expression of Z-linked genes is substantially reduced to that of autosomes [33]. But, from an evolutionary perspective, there is no currently available hypothesis to explain this reduced Z : A ratio in these species. The mechanisms of DC evolution can provide insights to answer this. It has been proposed that DC has evolved in a two-step process (Ohno's hypothesis) in *Caenorhabditis* and mammals [10–14], whereas in *Drosophila*, it is a direct upregulation of X-linked genes by DC complex (DCC) in males (XY) [8,9] (see Introduction). We propose that the basic element of driving force for the evolution of such diverse mechanisms with a single objective of achieving DC should be the ‘ancestral expression of proto-sex chromosomes’. Here, the ancestral expression refers to the fixed point of average gene expression for proto-sex chromosomes in the course of sex chromosome evolution. For instance, in *Drosophila*, *Caenorhabditis* and mammals, the value of ancestral expression of proto-X might be fixed as 1 [10–14]. So, in males (XY), the expression level of X is 1 and in females (XX) it is 2. Hence, in these species, the DC force would have evolved by choosing one of the possible/suitable paths like ‘DCC-mediated X upregulation’ or ‘two-step process’ (Ohno's hypothesis) to attain the destined expression level of 2 for X chromosome(s) in males XY = 2 and females XX = 2, which matches the value of autosomes (AA = 2). If the ancestral expression of proto-X/Z gets fixed as approximately 0.6 or so in a species, then the DC evolutionary pressures may choose a different path like, ‘repression of ZZ expression in homogametic sex’ for achieving DC. For example, in the case of *B. mori*, the Z : A ratios were approximately 0.6 in both sexes (table 1), but the M : F ratios for both A- and Z-linked genes are approximately 1, representing the equally expressing A- and Z-linked genes in both sexes (electronic supplementary material, table S4). Based on these observations, we speculate that the DC evolutionary pressures in *B. mori* would have selected for the repression of Z in homogametic sex, in order to achieve the DC faster. Thus, in short, the ‘ancestral expression of proto-sex chromosomes’ is one of the crucial determinants of the X : A or Z : A ratio, which in turn drives the DC evolutionary pressures to choose the path of DC mechanism (ancestral expression of proto-sex chromosomes > X : A or Z : A ratio > DC path).

### 3.2. Sex-biased expression of Z at early embryonic stages

The M : F ratio distributions imply the sex-biased expression of genes for the samples. In our study, these ratios suggest a clear male-biased expression of Z-linked genes at 96 h and a female-biased expression at 120 h (table 3). A stark transition of Z from a male-biased expression at 96 h to a female-biased expression at 120 h probably indicates the onset of DC effect probably initiated after 96 h and executed at the later stage, 120 h of development. A significant male-biased expression of A- and Z-linked genes being observed at the early stages of 78 and 96 h gets normalized at a later stage of 120 h, suggesting the advent of DC mechanism. This scenario clearly indicates the primary objective of DC mechanism to equalize the expression differences of A- and Z-linked genes between sexes [20]. In head samples, Z-linked genes showed an almost unbiased expression suggesting its compensation. By contrast, a slightly male-biased expression (statistically significant, table 3) of Z at 78 h stage may be initial, and we presume that this stage might not be representing a relative full-fledged Z-linked gene expression, based on comparatively lower Z : A ratios at 78 h stage (figure 2 and table 2).

However, from the heatmap, it is evident that a large number of genes with higher expressions showed a male-biased expression at 78 and 96 h stages. These genes shift from a male-biased to -unbiased expression (based on similar colour schema observed between sexes) at the later stage of 120 h. This indicates the advent of dosage compensatory effect over these genes at this stage and also suggests that the onset of DC can be considered to initiate post 96 h. In the head samples, except for a very small subset of genes, almost all the genes showed a comparable level of expression, indicating the established DC. Although there is only evidence of Z suppression in males [43], the clue obtained from the observation of a relatively increased expression of genes with higher expression (figure 7, cluster 9) in females, compared with male embryos at 120 h stage, suggests the existence of Z hyperactivation in females. At 78 and 96 h stages, the genes with lower expression (figure 7, cluster 2) were female-biased and turned out to be male-biased at the later stage of 120 h stage on an overall basis, suggesting an inverse effect of DC mechanism over this subset (lower expression, figure 7, cluster 2) of genes.

From this study, we have identified an embryonic stage (120 h), at which the effect of DC comes into action (figure 2). Gene-wise comparisons at 96 h showed a profound male-biased Z-linked gene expression, which gets counter-attacked by DC at 120 h, exhibiting a slightly female-biased Z-linked gene expression at this stage. The male-biased expression of Z-linked genes in the early stages of development (78 and 96 h) could be due to the functionally inactive, putative DCC whose initiation (post 96 h) and advent from the later stage (120 h) would have established the DC in *B. mori* tissues. We suggest 96 h as a crucial developmental stage both for DC, based on relatively higher expression of *masc*, a dosage regulator gene in males (electronic supplementary material, table S2) and also in sex determination, due to the initiation of *dsx* sex-specific differential splicing (Ajimura M. *et al*. 2017, unpublished data; [48]). From our results, the complete DC is apparent in *B. mori*, depicting its essentiality for sexual fitness in this species. The complete DC is represented by an overall Z-linked gene expression parity between the sexes with a relatively reduced expression compared with autosomes, a unique trend generally not seen in the dosage-compensated taxa [12]. This type of reduced expression of ZZ in *B. mori* males is analogous to the downregulation of both XX transcription in hermaphrodite *C. elegans*, probably by increased chromosome condensation [49]. The speculated DC mechanism of *C. elegans* promoted by *XO lethal-1* (*xol*-*1*) is similar to that of the *masc* [43] in the *B. mori*; both the genes regulate the sex determination and the DC. Both the dosage mechanisms result in the hypoexpression of the XX/ZZ chromosomes.

### 3.3. Dosage compensation occurs through reduced expression or hyperexpression of Z chromosome?

The emerging evidence suggests that the patterns of DC are highly variable across sex-determination system and species [14]. The DC observed in *B. mori* stands as a unique mechanism, which is achieved mostly through the hypoexpression of the ZZ chromosomes in males. In a few ZW species studied, male ZZ : AA expression ratios in general were reported to be approximately 1, e.g. in Aves it is 1.01 [27,28], in nematode *Schistosoma mansoni* it is 1.06 [30] and in lepidopteran *P. interpunctella* it is 0.95 [32]. Whereas, in females ZW/AA ratios have been reported to be approximately 0.5 (table 4), suggesting an incomplete DC in these species. In *Heliconius* species, the DC is reported to be imperfect or incomplete based on a consistent male-biased expression of Z-linked genes in various tissues tested for dosage [33]. By contrast, these ratios were almost equal between the sexes and have a value of less than 1 in the lepidopterans *M. sexta* (ZZ : AA = 0.83 and ZW/AA = 0.81) [31] and *B. mori* [ZZ : AA = approximately 0.6 (mean) and ZW/AA = approximately 0.6 (mean)], suggesting the complete DC (table 4).

**Table 4.** Comparing the Z : A and M : F ratios between dosage-investigated ZW species.

ZW species (complete dosage present)	ZZ/AA	ZW/AA	A-M/F	Z-M/F
*Bombyx mori* (head and 120 h) (current study)	0.62 and 0.63	0.64 and 0.64	0.99 and 1.07	0.97 and 1.04
*Manduca sexta*	0.83	0.81	0.98	1

ZW species (complete dosage absent)	ZZ/AA	ZW/AA	A-M/F	Z-M/F
*Plodia interpunctella*	0.95	0.53	0.96	<1.5
*Aves*	1.01	0.67	0.99	1.4
*Schistosoma*	1.06	0.58	1.13	2.06

The median Z : A ratios (figure 2) suggest that in male embryos, the Z-linked gene expression reaches the relative expression point of approximately 0.5 first compared with females (observed first at 96 h stage). But at a later stage of development (120 h), the Z-linked gene expression was found to be slightly higher in females compared with males (figure 2), contrasting the double dose (copy number) of Z chromosomes in males. This can be explained by these possibilities: (i) the hyperexpression of single Z chromosome in females, (ii) by the hypoexpression of ZZ in males, or (iii) by a combinatorial effect of both hyper- and hypoexpressions of Z-linked genes.

A recent report suggests the involvement of a Z-linked CCCH type zinc finger gene, *masc* in the DC, as the RNAi of *masc* resulted in upregulation of Z-linked genes [43]. In this study, we observed a *masc* gene expression level-driven suppression of Z-linked genes in males (ZZ)*.* The overall Z-linked gene expression difference between sexes at 96 h was found to be significantly different (figure 1). The *masc* gene at 96 h stage was found to be male-biased (electronic supplementary material, table S2). Being hypothesized to be involved in the mechanism of DC, the increase in the fold change of *masc* at 96 h suggests its crucial involvement in the hypoexpression of ZZ in males to that of the Z in females [43]. The male-biased expression of *masc*, especially at the dosage uncompensated stage of 96 h, denotes its probable involvement in the mechanism of DC, and this male-biased *masc* expression would have led to the hypoexpression of ZZ at the immediate next embryonic stage of 120 h where the Z dosage was compensated (figure 6). As the *masc* gene was also shown to be involved in the mechanism of DC by suppression of ZZ in males, its male-biased expression at 96 h stage of development (electronic supplementary material, table S2) probably suggests its crucial involvement in DC [43].

The overall Z-linked gene expression is slightly female-biased at 120 h stage (figure 7), this may be due to ‘dosage over-compensation effect’, an exact opposing phenomenon to ‘inverse dosage effect’, observed in *D. melanogaster* in which there is a upregulation of single X chromosome in males due to loss of repressors [50,51]. We presume this effect as temporary, mediated by the gain of ZZ repression in males due to a tight transcriptional downregulation of the freely expressing ZZ chromosomes in males [43]. Besides, this effect may also be treated as over-representation of single female Z chromosome expression in the background of ZZ repression in males. Because of this effect, we presume that the freely expressing female Z chromosome at 120 h stage seems to be apparently higher than its male counterpart. The M : F ratio distributions (figure 5) of A and Z clearly indicate the initial absence (96 h) and probable establishment of DC in the later stage (120 h) of embryo development. Based on this finding, we speculate that the reason for lower Z : A ratio observed in 120 h male could be due to the suppressed expression of Z chromosome at this stage; high level of *masc* expression at 96 h may be suppressing ZZ expression in males, thereby bringing down the Z expression at 120 h stage (table 2). All these findings suggest and support the hypothesis of probable suppression of Z-linked genes in males [43].

Furthermore, there could be two possibilities of suppression of Z-linked gene expression in males: (i) by suppression of both the Z-linked loci in males; (ii) by inactivation of one of the Z chromosomes similar to the hetero-chromatinization of one of the X chromosomes by bar body formation in mammals [20]. We strongly believe in the possibility of suppression of both the Z-linked loci in males based on indirect evidence that no Barr bodies were identified in *B. mori* histology.

Additionally, the lepidopterans studied for DC showed mixed patterns of complete (*M. sexta* and *B. mori*), incomplete (*P. interpunctella*) and imperfect (*Heliconius*) types of DC mechanisms. Generally, it is believed that the Z chromosome synteny (scaffold or contig order) is conserved among lepidopterans [52,53]. However, several distinctly unique micro-syntenies (gene constitution) or sex chromosomal fusions may be present in various species. For example, in a few lepidopterans like *Samia cynthia pryeri*, the Z chromosome is fused with the 12th and 13th chromosomes suggesting the existence of neo-chromosome of Z chromosomes [54], and in *Bombyx* the Z chromosome is enriched with testis-specific genes [34]. Similarly, several unknown differences may be naturally occurring among the lepidopteran Z chromosomes. These structural variations in sex chromosomes with respect to gene constitution might ultimately influence the DC evolutionary forces to choose different paths, thus yielding differential patterns of DC in lepidopterans. It is interesting to note that in *Drosophila*, a male heterogametic species, DC is achieved by the hypertranscription of single X chromosome in males [8], and in *Bombyx*, a female heterogametic species, the DC is achieved by the hypo/reduced transcription of two ZZ chromosomes in males [43]. This suggests that the DC evolutionary force has chosen the counteracting/opposite mechanisms in the male and female heterogametic species for achieving the DC.

Altogether the growing data and analyses, especially using RNA-seq, in various species present a dynamic picture of patterns of DC, suggesting the initial selection of highly diverse mechanisms being adapted by the evolutionary forces with a focused objective of achieving the DC. A re-evaluation of DC in mammals and *C. elegans* using RNA-seq data contradicts Ohno's hypothesis, questioning our current knowledge of the sex chromosome evolution and DC mechanisms [55]. Hence, by taking the advanced and accurate RNA-seq data into consideration, there is a necessity of critical revision of current evolutionary theories on DC. A new theory should emerge in order to explain the reasons for lower expression of X or Z to that of autosomes to gain a comprehensive understanding of the sex chromosome evolution and DC mechanisms.

## 4. Conclusion

In this study, we have compared dosage of Z-linked genes in different embryonic stages between male and female sexes and showed that Z-linked gene expression dosage was not compensated in early embryonic stages. As the embryo ages and after the upregulation of *masc* gene, Z-linked genes in males show a lower expression, compensating with the dosage of females. We speculate that DC emerges after 96 h in male silkworm. In the embryo samples, 96 h is considered as a crucial developmental stage, at which the sex determination and differentiation are more finely tuned in the embryos. To our knowledge, this is the first report showing the initiation of DC in embryonic stages using RNA-seq data. We also show complete DC in the larval stage of *B. mori* by comparing male and female transcriptomes of the head tissue. In *B. mori*, the type of complete DC observed is ZZ : AA = Z : AA < 1, which is very distinct to that of XX/AA = X/AA = 1. Further studies have to be conducted to confirm whether this compensation is through silencing of one of two Z chromosomes in males or through the reduced expression of genes in both the copies of Z chromosome.

## 5. Material and methods

### 5.1. Sample collection, library preparation and RNA-sequencing

Two W-chromosome mutant strains of *B. mori* were used: (i) Japanese sex limited (JPSL) for the sexed embryo collection and (ii) sex-limited strain (QGSLO) for the sexed larval heads. In JPSL, the translocation of chromosome 10 fragment harbouring the *kynurenine monooxygenase* gene on to the W-chromosome is believed to be responsible for the development of dark brownish serosal pigmentation, which acts as a visible marker to differentiate female embryos as early as 36 h. In the QGSLO strain, female larvae can be easily distinguished by distinct crescent-shaped markings on the dorsal side of larva from the fourth-instar stage. For the embryo collection, JPSL strain moths were set for 4 h mating at room temperature, transferred to 4°C overnight, followed by depairing and were set in dark at room temperature for 2 h for a uniform egg laying. Collected eggs were cold acid-treated (one of the methods of breaking diapause) at an age of 20 h to break the diapause and were thoroughly washed under running tap water and incubated at 25°C for the development to proceed. At 36 h, male and female eggs were segregated based on the serosal pigmentation. Sampling of 200 each of male and female embryos was done at 78, 96 and 120 h. For the head tissue collection, larvae of (QGSLO) fifth-instar 5th day were numbed on ice for 30 min, decapitated, 10 male and 10 female larval heads were pooled and snap-frozen in liquid nitrogen until use.

For collecting BmN cells, log-phase cells that are regularly passaged in TC-100 (Sigma) insect media with 10% FBS (Gibco) were selected, slogged, pelleted in PBS and stored at −80°C until use. From the collected samples, total RNA was isolated and on-column DNAseI treated for the removal of genomic DNA contamination using the Direct-Zol RNA isolation kit (Zymo Research). RNA libraries were prepared following the TruSeq RNA sample preparation kit v2 (catalogue no. RS-122–2001) from Illumina using 1 µg of mRNA. Sequencing was performed on an Illumina 1000 HiSeq platform (C-CAMP, Bangalore). In total, not less than approximately 60 million pair-end reads of 100 bp, for each of the sexed embryonic stages and head samples, were generated.

### 5.2. RNA-sequencing read quality filtration, mapping and data analyses

The RNA-seq read quality was assessed using the package FastQC [56]. The adapter removal and quality trimming was performed using Trimmomatic v. 0.35 [57]. The leading and trailing low quality or N bases were removed below quality 3, reads were scanned with a 4-base sliding window and clipped when the average quality per base dropped below 20, and read sequences below 30 bases in length were dropped. The filtered paired-end reads were mapped against the *B. mori* genome sequence and its annotations (downloaded from Ensembl release 29 (2015) (GCA_000151625.1.29) [58]) using Bowtie 2 v. 2.2.6 [59,60] with default parameters. The aligned reads were filtered to keep only uniquely mapped reads. SAM/BAM conversions, sorting, indexing and filtering were performed with SAMtools v. 1.2. [61]. The alignment files (SAM format) so obtained were imported into Seqmonk software [62]. While importing the SAM files into Seqmonk, the libraries were treated as pair-end and duplicate reads were eliminated. The log_2_-transformed FPKMs for genes were quantitated, by correcting for DNA contamination, transcript isoforms were merged, transcript length correction was made, besides excluding the genes with no or very low read counts (considered as noise in Seqmonk) to avoid bias in the data that might skew the analysis. The raw read counts table and quantitated genes report (FPKM values) of Seqmonk analysis were exported to Excel (Microsoft) and further analysed. Moreover, for *B. mori*, as the GFF file from Ensembl was not annotated with the chromosome number, which is required for current analysis. So, to overcome this, the ‘description’ code of each gene in the GFF file was replaced with its corresponding chromosome number, based on the scaffold identity from KAIKObase [63] annotation data using custom shell scripts.

### 5.3. Statistics for Z dosage and data representation

Based on the scaffold mapping, the genes were grouped into autosomal (A)- and Z-linked (Z) genes. The unmapped genes were excluded from further analysis. Similar to a conventional dosage analysis, the dosage in *B. mori* was tested by two estimates. They are (i) Z : A ratios (autosomal relative expression of Z-linked genes) and (ii) M : F ratios (sex-biased expression of A and Z-linked gene expressions). To assess the Z dosage effects in the samples, the ratio of autosomal and Z-linked gene expression (Z : A) was calculated within each sample (male and female), and this ratio was compared between the sexes. The true expression (FPKM ≠ 0) of all the genes were estimated by choosing the option, ‘Don't quantitate probes with no counts’ (probes = mRNA in our analysis) in Seqmonk during probe quantitation. The Z : A ratios were estimated using this ‘true expression dataset’ (FPKM ≠ 0) and resulted in mapping of approximately 10 000 genes for embryo samples and approximately 7000 genes for head samples. The mean and median Z : A ratios were estimated, which represent the relative expression level of Z-linked genes compared with that of autosomal. Bootstrapping (10 K) of log-transformed FPKM data was performed to find the 95% confidence interval for the Z : A point estimate of the median, using the online web tool STATKEY [64]. We compared the median level expression differences between autosomal and Z-linked genes within the sex for each sample individually by non-parametric MWU test (Wilcoxon rank-sum test), conducted in R package [65]. Furthermore, the M : F ratio distributions were calculated from (i) FPKM values (electronic supplementary material, file S1) and (ii) raw counts data (electronic supplementary material, files S2–S5) of genes that showed expression (FPKM ≠ 0) in both sexes. Furthermore, the genes with less than four raw read counts in at least one of the samples out of four (comparison: 2 male technical replicates × 2 female technical replicates) were removed and the remaining genes were subjected to the trimmed mean of M-values (TMM) normalization method (electronic supplementary material, file S6 and for script electronic supplementary material, file S7) for the estimation of M : F ratio distributions [66]. The log_2_ (M : F) density distributions were generated using Wessa.net histogram, online web tool [67] to reveal the overall picture of the sex-biased distribution of the autosomal and Z-linked genes in the samples. The median level differences between the M : F distributions for autosomal and Z-linked genes were also tested by the MWU test. To compare and explore any discrepancies found between male and female median Z-linked genes at various levels of gene expressions, an unpaired comparison for the Z-linked gene expression data between sexes was conducted. For this, the Z-linked log_2_ FPMK distribution data for both the sexes were sorted independently in a descending order and were divided into quartiles (Q1–Q4), each representing different magnitudes of gene expression (high—Q4, medium—Q3, low—Q2 and very low—Q1 expressing genes) (figure 3; for script, electronic supplementary material, file S7). For the quartile-based max of male or female data analysis, the Z-linked genes were segregated as paired data and split into quartiles (figure 6; for script, electronic supplementary material, file S7). The median expression difference within quartiles between sexes was tested by the MWU test. Additionally, to view the profile of Z-linked gene expression, all the Z-linked genes were filtered and saved as annotation track from which a heatmap was generated based on clustering. For generating heatmap in Seqmonk, even the loci with no true expressions were also quantitated.

### 5.4. cDNA preparation

The RNA concentration of all the samples was measured using Nanodrop 2000 (Thermo Scientific). cDNA was synthesized from 1 µg of total RNA using SuperScript™ III (Invitrogen), following the manufacturer's instructions. Briefly, 1 µg of total RNA, 1 µl of 10 mM dNTPs, 1 µl of 12 nucleotide oligodT primer and dH_2_O to 13 µl were added in a tube and incubated at 65°C for 5 min, followed by freezing the mixture on ice for 1 min. To this mixture, 1 µl of 0.1 M dithiothreitol, 4 µl of 5× buffer, 1 µl of SuperScript enzyme and 1 µl of RNaseOut were added, mixed with pipette and incubated at 50°C for 1 h followed by stopping the reaction at 75°C for 15 min.

### 5.5. Quantitative RT-PCR

The relative expression of selected genes (see electronic supplementary material, table S5 for primer sequences) was validated through qRT-PCR (ABI 7500). The reaction was set using SYBR Premix Ex Taq (Tli RNaseH Plus) from Takara Bio, Inc. The reaction mixture included cDNA sample of 3 µl (diluted to 10 ng µl^−1^), 0.2 µM primers in a final volume of 20 µl of master mix. Reaction conditions were: 95°C for 30 s, 95°C for 5 s and 60°C for 34 s. The standard curve analysis was done using ABI SDS software, v. 1.2.3. The reactions were carried out in triplicates and the relative expression was determined using Δ*C*_t_ analysis. Fold change values for male samples relative to female samples (calibrator) were obtained by normalizing the gene expression values to the *rp49* as endogenous/internal reference control separately [47].

## Supplementary Material

Supp Tables

## Supplementary Material

Supp File 1

## Supplementary Material

Supp File 2

## Supplementary Material

Supp File 3

## Supplementary Material

Supp File 4

## Supplementary Material

Supp File 5

## Supplementary Material

Supp File 6

## Supplementary Material

Supp File 7

## Data accessibility

All sequence data are available through BioProject ID: PRJNA388026.
