# Correction to ‘RNA sequencing reveals a complete but an unconventional type of dosage compensation in the domestic silkworm *Bombyx mori*’

**DOI:** 10.1098/rsos.171023

**Published:** 2017-08-30

**Authors:** Gajula Gopinath, Kuchi Srikeerthana, Archana Tomar, Srikakolapu M. Ch. Sekhar, Kallare P. Arunkumar

*R. Soc. open. sci.* **4**, 170261. (Published 12 July 2017). (doi:10.1098/rsos.170261)

The Authors' contributions section in the published paper is incorrect. The correct authors' contributions are as follows:

K.P.A. conceived and designed the project and interpreted the results. G.G. prepared the samples for the study, conceived and designed the approach, analysed the data, interpreted the results and wrote the manuscript. S.K., A.T. and S.M.Ch.S. analysed the data and interpreted the results. K.P.A., S.K., A.T. and S.M.Ch.S. contributed in writing and improving the manuscript. All authors have read and approved the final version.

