# Supplementary material for: RNA sequencing reveals a complete but an unconventional type of dosage compensation in the domestic silkworm Bombyx mori
Source: R Soc Open Sci. 2017 Jul 12;4(7):170261. doi: 10.1098/rsos.170261 (PMC5541547; doi:10.1098/rsos.170261)
Supplement: Supp Tables [file rsos170261supp1.docx]

**Supplementary Tables**

Table S1: Number of paired reads before and after Trimmomatic

| **Stage**, Sample | Total  paired end reads | paired end reads after trimming |
| --- | --- | --- |
| **Embryonic, 78h** |  |  |
| Male, technical replicate 1 | 11050822 | 9914362 |
| Male, technical replicate 2 | 31628333 | 30890006 |
| Female, technical replicate 1 | 16233927 | 15815447 |
| Female, technical replicate 2 | 2697763 | 2443184 |
|  |  |  |
| **Embryonic, 96h** |  |  |
| Male, technical replicate 1 | 13884150 | 13260716 |
| Male, technical replicate 2 | 26784132 | 26457089 |
| Female, technical replicate 1 | 13342408 | 12649208 |
| Female, technical replicate 2 | 16435822 | 16235564 |
|  |  |  |
| **Embryonic, 120h** |  |  |
| Male, technical replicate 1 | 17455056 | 16955766 |
| Male, technical replicate 2 | 17454043 | 16746448 |
| Female, technical replicate 1 | 13499099 | 12938869 |
| Female, technical replicate 2 | 20835824 | 20603602 |
|  |  |  |
| **Larval, Head** |  |  |
| Male | 49965704 | 43855957 |
| Female | 48243611 | 43641574 |
|  |  |  |
| **BmN cells** |  |  |
| Biological replicate 1 | 28818725 | 24947548 |
| Biological replicate 2 | 30766050 | 25902378 |
|  |  |  |
| **Total =** | **359095469** | **333257718** |
|  |  |  |

Table S2: Expression of *masc* gene in the embryonic and head samples.

| **Sample** | ***Masc* FPKM in males** | ***Masc* FPKM in females** | ***Masc* gene M/F Fold Change** |
| --- | --- | --- | --- |
| 78h | 0.255 | 0 | -- |
| 96h | 0.92 | 0.14 | 6.45* |
| 120h | 2.01 | 2.11 | 0.95 |
| Head | 0.67 | 0 | -- |

**masc* gene showed male biased expression at 96h, speculated to be a crucial stage for sex determination and DC. M=male and F=female.

Table S3: Comparison of Z expression level to that of autosomes, from Figure 7

|  | **Chromosomes with higher average expression than Z (12)** | **Chromosomes with lesser average expression than Z (7)** | **Chromosomes with equal average expression as Z (8)** |
| --- | --- | --- | --- |
| **Chromosome number** | 3,4,5,8,9,10,11,13,15,17,18 & 22 | 2,7,14,20,26,27 & 28 | 6,12,16,19,21,23,24 & 25 |
| **Size in Mb** | **201.83** | 75.43 | 122.37 |
| **% of genome size (419.98 Mb)** | 48.06 | 17.96 | 29.14 |

The autosomes were grouped in to three, based on their relative Z expression level (Figure 7) and their percentages of genomic sizes were calculated to show that a major proportion of genome show on an average higher expression than that of Z chromosome.

Table S4: The male to female Autosomal and Z expression ratios (M:F) for the head samples.

|  | **Autosomal (7210 genes)** | **Z linked**  **(339 genes)** |
| --- | --- | --- |
| **Head (median, M:F ratio)** | 0.904* | 1.019* |
| **Median FPKM (Male/Female)** | 4.4969/4.973 | 2.8172/2.763 |

*These ratios of ~1 represent an equal expression of the autosomal and the Z linked genes between sexes.

Table S5: The list of primer sequences used for their qRT-PCR analysis of selected genes.

| **Sc.No** | **Primer_Name** | **Primer** |
| --- | --- | --- |
| 1 | BGIBMGA000127_F | GCATCTAGCACAGACAGTGA |
| 2 | BGIBMGA000127_R | ATCTGACATCGTTAATACCA |
| 3 | BGIBMGA003212_F | TGAAACACCCGTTTGGCAAG |
| 4 | BGIBMGA003212_R | TAGCTTGTTCCTTGAACGCC |
| 5 | BGIBMGA000971_F | ACAAGACCTAAACTAGGTTT |
| 6 | BGIBMGA000971_R | CAAGTGATCTCTCTATGACA |
| 7 | BGIBMGA000138_F | GGGCTTGGAGGAACAACATA |
| 8 | BGIBMGA000138_R | ACCTGCATCACTGCCGTCAA |
| 9 | BGIBMGA002917_F | AAGTCCCTGCAACGAAATTA |
| 10 | BGIBMGA002917_R | CTCCGCCCGGTATTATCCTA |
| 11 | BGIBMGA002150_F | AAAGCAGAGCTTGCTGTAAA |
| 12 | BGIBMGA002150_R | CACTTTCCTCTTCTATATCG |
| 13 | BGIBMGA000608_F | CTAAATATGTGGAGGACCTTAT |
| 14 | BGIBMGA000608_R | CATCATATATAAAGTAGCGAAG |
| 15 | BGIBMGA000721_F | ATACGTTGTGTACGCGCCGCTG |
| 16 | BGIBMGA000721_R | CGGCTTGGCTGTGGTACTACAC |
| 17 | BGIBMGA002146_F | TGAACTACCAGTAAATTAC |
| 18 | BGIBMGA002146_R | AAGACAGCTAGTTAAATAG |
| 19 | BGIBMGA003852_F | GCCACCGAGAACATGAAAGCT |
| 20 | BGIBMGA003852_R | TCTTTCTTCCGTGACGACCAG |
| 21 | BGIBMGA001500_F | ATTGTCAACCAGGAGGTGAG |
| 22 | BGIBMGA001500_R | TCCAGTTGGTCAACGCTCAA |
