# Supplementary material for: RNA sequencing reveals a complete but an unconventional type of dosage compensation in the domestic silkworm Bombyx mori
Source: R Soc Open Sci. 2017 Jul 12;4(7):170261. doi: 10.1098/rsos.170261 (PMC5541547; doi:10.1098/rsos.170261)
Supplement: Supp File 7 [file rsos170261supp8.docx]

**R-script for TMM Normilization and edgeR analysis (with FDR cutoff 0.05)**

Reference: edgeR: differential expression analysis of digital gene expression data, User’s Guide, Yunshun Chen, Davis McCarthy, Matthew Ritchie, Mark Robinson, Gordon K. Smyth.

library(edgeR) #loding edgeR

# Importing data

a <- read.csv("/Path/to/the/file/Raw_counts.csv", header = T, sep="\t", check.names=FALSE)

head(a)

rownames(a) <- a$Probe_Description

# Removing columns of other time points

a[1] <- NULL

head(a)

a[5:16] <- NULL

head(a)

# Grouping of male and female || duplicates (replace 78 with other time points as necessary)

gro <- c(rep("78F",2), rep("78M",2))

# Creating DEGList object

a1 <- DGEList(counts = a, group =gro)

a1

# Filtering reads with CPM less than 4 for 2 samples

keep <- rowSums(cpm(a1)>4) >= 2

table(keep)

a2 <- a1[keep, , keep.lib.sizes=FALSE]

# Normaliztion

a3 <- calcNormFactors(a2, method="TMM")

a3

a3$samples

#write out Normalization factors and filtered data

write.csv(a3$counts, file="78H_filtered_dataset.xls", quote = FALSE)

write.csv(a3$samples, file="78H_Norm_factor_filtered.xls", quote= FALSE)

# Data Exploration

plotMDS(a3)

b1 <- estimateCommonDisp(a3)

b2 <- estimateTagwiseDisp(b1)

#exact test

et <- exactTest(b2)

etp <- topTags(et, n=300000)

summary(etp$table)

etp$table$logFC = -etp$table$logFC

pdf("edgeR-MA_78_Normalized-plot.pdf")

plot(

etp$table$logCPM,

etp$table$logFC, main="78hrs MA Plot",

xlab="logCPM", ylab="logFC",

xlim=c(-3, 20), ylim=c(-12, 12), pch=20, cex=.3,

col = ifelse( etp$table$FDR < .05, "red", "black" ) )

abline(h=c(-1,1), col="blue")

dev.off()

# output CSV

write.csv(etp$table, "edgeR-78M_vs_78F_Normalized.csv", quote=FALSE)

**R-script for generating Quantile analysis for Unpaired data Figure 3**

# Remove all variables and get input.csv data

rm(list=ls(all=TRUE))

source("/home/keerthana/Desktop/Quartile_analysis/Gopinath/FPKM_QUARTILE/NEW/Qbox.R")

dat <- read.csv("FPKM_Unpaired_changed.csv",header=TRUE,sep = ",")

#head(dat)

# Create output directory

outdir <- "/home/keerthana/Desktop/Quartile_analysis/Gopinath/FPKM_QUARTILE/NEW/"

# Setup plotting parameters

png(filename = paste(outdir, "unpaired_all_in_one.png"), units = "in", w= 15, h = 6, res = 300)

par(mfrow = c(1,4))

yLim = c(-4,8)

Qbx(male = log(dat$X78Male),female = log(dat$X78Female), ylim = yLim, crit = "pmax", sub = "Quartile_max", main = "Quartiles based max of \n 78h male or female data"  )

Qbx(male = log(dat$X96Male), female = log(dat$X96Female), ylim = yLim, crit = "pmax", sub = "Quartile_max", main = "Quartiles based max of \n 96h male or female data"  )

Qbx(male = log(dat$X120Male), female = log(dat$X120Female), ylim = yLim, crit = "pmax", sub = "Quartile_max", main = "Quartiles based max of \n 120h male or female data"  )

Qbx(male = log(dat$Head_Male), female = log(dat$Head_Female), ylim = yLim, crit = "pmax", sub = "Quartile_max", main = "Quartiles based max of \n head male or female data"  )

dev.off()

**R-script for generating Quantile analysis for Paired data Figure 6**

# Remove all variables and get input.csv data

rm(list=ls(all=TRUE))

source("/home/keerthana/Desktop/Quartile_analysis/Gopinath/FPKM_QUARTILE/NEW/Qbox.R")

dat <- read.csv("FPKM_newpaired_changed.csv",header=TRUE,sep = ",")

#head(dat)

# Create output directory

outdir <- "/home/keerthana/Desktop/Quartile_analysis/Gopinath/FPKM_QUARTILE/NEW/"

# Setup plotting parameters

png(filename = paste(outdir, "paired_all_in_one.png"), units = "in", w= 15, h = 6, res = 300)

par(mfrow = c(1,4))

yLim = c(-4,8)

Qbx(male = log(dat$X78Male),female = log(dat$X78Female), ylim = yLim, crit = "pmax", sub = "Quartile_max", main = "Quartiles based max of \n 78h male or female data"  )

Qbx(male = log(dat$X96Male), female = log(dat$X96Female), ylim = yLim, crit = "pmax", sub = "Quartile_max", main = "Quartiles based max of \n 96h male or female data"  )

Qbx(male = log(dat$X120Male), female = log(dat$X120Female), ylim = yLim, crit = "pmax", sub = "Quartile_max", main = "Quartiles based max of \n 120h male or female data"  )

Qbx(male = log(dat$Head_Male), female = log(dat$Head_Female), ylim = yLim, crit = "pmax", sub = "Quartile_max", main = "Quartiles based max of \n head male or female data"  )

dev.off()
